# Association of Serum Albumin With Apnea in Infants With Bronchiolitis: A Secondary Analysis of Data From the MARC-35 Study

**DOI:** 10.1001/jamanetworkopen.2019.7100

**Published:** 2019-07-17

**Authors:** Jonathan M. Mansbach, Ruth J. Geller, Kohei Hasegawa, Janice A. Espinola, Michelle D. Stevenson, Ashley F. Sullivan, Carlos A. Camargo

**Affiliations:** 1 Department of Pediatrics, Boston Children's Hospital, Boston, Massachusetts; 2 Department of Emergency Medicine, Massachusetts General Hospital, Harvard Medical School, Boston; 3 Department of Pediatrics, Emergency Medicine, Norton Children’s Hospital, University of Louisville School of Medicine, Louisville, Kentucky

## Abstract

**Importance:**

Apnea is a rare, life-threatening complication of bronchiolitis, the leading cause of infant hospitalization in the United States. Currently, no objective method exists for identifying which infants will become apneic.

**Objective:**

To investigate whether serum albumin levels are associated with apnea in infants with severe bronchiolitis.

**Design, Setting, and Participants:**

A secondary data analysis of the 35th Multicenter Airway Research Collaboration, an ongoing multicenter cohort study of infants hospitalized for bronchiolitis, was conducted from December 11, 2018, to May 30, 2019. Seventeen hospitals across the United States enrolled infants (n = 1016) during 3 consecutive bronchiolitis seasons (November 1 to April 30) between 2011 and 2014. Infants with heart-lung disease or a gestational age less than 32 weeks were excluded.

**Exposures:**

Serum albumin level was categorized as low (<3.8 g/dL) or normal (≥3.8 g/dL).

**Main Outcomes and Measures:**

Apnea during the hospitalization.

**Results:**

Of the 1016 infants hospitalized for bronchiolitis, the median (interquartile range [IQR]) age was 3 (2-6) months, 610 (60.0%) were male, and 186 (18.3%) were born preterm (32-37 weeks’ gestation). Among the 25 infants (2.5%) with apnea while hospitalized, the median (IQR) serum albumin level was 3.5 (3.1-3.6) g/dL, and 22 (88.0%) had low serum albumin levels. The prevalence of apnea was 5.7% among all infants with low albumin levels, compared with 0.5% prevalence in infants with normal serum albumin levels. In unadjusted analyses, apnea was associated with younger age, preterm birth, weight-for-age *z* score, and low albumin (odds ratio [OR], 12.69; 95% CI, 3.23-49.82). After adjustment for age, preterm birth, and weight-for-age *z* score, low serum albumin levels remained statistically significantly associated with apnea (OR, 4.42; 95% CI, 1.21-16.18).

**Conclusions and Relevance:**

Low serum albumin levels appeared to be associated with increased risk of apnea after adjustment for known apnea risk factors. This finding provides a path to potentially identifying apnea, a life-threatening complication of bronchiolitis.

## Key Points

**Question:**

Is serum albumin level associated with the risk of apnea in infants with bronchiolitis?

**Findings:**

In this secondary analysis of a cohort of 1016 infants hospitalized for bronchiolitis, low serum albumin was statistically significantly associated with a higher risk of apnea during the hospitalization. This association was independent of known apnea risk factors (younger age, premature birth, and weight-for-age *z* score).

**Meaning:**

Albumin levels may have a role in identifying apnea in bronchiolitis.

## Teaser

This secondary analysis of the 35th Multicenter Airway Research Collaboration cohort study investigates whether serum albumin levels are associated with apnea in infants hospitalized for bronchiolitis.

## Introduction

Infants with bronchiolitis usually have mild symptoms, but every year in the United States approximately 130 000 hospitalizations, 50 known deaths, and an unclear number of sudden unexplained deaths among infants occur are associated with bronchiolitis.^1,2,3^ One of the potentially life-threatening complications of bronchiolitis is apnea.^4^ The incidence of apnea among infants with bronchiolitis varies by the population under study. Retrospective and prospective studies over the past 20 years have found the incidence of apnea to range from 1% in healthy term infants to 17% in preterm infants.^5^ Although risk factors have been identified for apnea (eg, young age, preterm birth), it remains unclear which infants with bronchiolitis will develop this rare outcome. As a result, clinicians try to balance the safety of infants who have bronchiolitis and are at risk for apnea with potentially unnecessary hospitalizations for observation.^4^

Albumin is a critical multifunctional serum protein that drives colloidal osmotic pressure, binds biologically important compounds (eg, medications, bilirubin, and vitamin D), and has antioxidant activity.^6^ Although the mechanism remains unclear, low serum albumin is associated with higher mortality risk in children and adults with several medical conditions, including respiratory illness.^7,8,9,10^

We conducted a secondary data analysis of the 35th Multicenter Airway Research Collaboration (MARC-35), a prospective, multicenter study of infants with severe bronchiolitis (ie, requiring hospitalization).^11^ Our goal for the present study was to examine the association between serum albumin levels and apnea in this population.

## Methods

The MARC-35 study, as described elsewhere,^11^ is an ongoing 17-center prospective cohort study of hospitalized infants (aged <1 year). The overall objective of MARC-35 is to examine the association between the characteristics of severe bronchiolitis and the risk of recurrent wheezing and asthma. All participants in MARC-35 have an attending physician’s diagnosis of bronchiolitis as defined by the American Academy of Pediatrics.^12^ This secondary analysis, conducted from December 11, 2018, to May 30, 2019, focused on infants who were enrolled across the United States during 3 consecutive bronchiolitis seasons (November 1 to April 30) from 2011 to 2014. Infants with heart-lung disease or a gestational age less than 32 weeks were excluded. The institutional review boards at the 17 enrolling hospitals approved the present study, and all participating families provided written informed consent.

Site teams extracted data from outpatient clinic, emergency department, and inpatient records. To complement these data, the teams also conducted structured interviews with parents or legal guardians while the infants were hospitalized.

Site teams used standardized equipment (Medline Industries) and followed a protocol^13^ to collect nasopharyngeal aspirates from all infants within 24 hours of hospitalization. Quantitative real-time reverse transcriptase–polymerase chain reaction was conducted at Baylor College of Medicine for respiratory syncytial virus (RSV) types A and B and 15 other viruses, as previously described.^14^ For the current analysis, the viral origin was categorized as RSV infection (ie, includes viral co-infections) or non-RSV infection.

In addition, site teams collected blood from all infants within 24 hours of hospitalization. The serum was tested for albumin using the albumin BCP assay (Abbott Laboratories). The primary exposure, serum albumin level, was dichotomized at the lower bound of the pediatric reference range and categorized as low (<3.8 g/dL) or normal (≥3.8 g/dL) (to convert albumin level to grams per liter, multiply by 10).

The primary outcome was the occurrence of apnea during the hospitalization. A secondary outcome was apnea occurring either before (emergency department or clinic) or during hospitalization. Both outcomes were obtained from medical record reviews (ie, by history or observation in the hospital). Undocumented presence of apnea was categorized as no apnea.

### Statistical Analysis

Bivariate analyses included the Kruskal-Wallis test, χ^2^ tests, and logistic regression. We assessed the distributions of albumin levels by presence of apnea using histograms. Multivariable logistic regression models were adjusted for age at hospitalization; preterm birth, defined as 32 to 37 weeks’ gestation; and weight-for-age *z* score at hospitalization. Weight-for-age *z* scores were calculated according to the 2006 World Health Organization child growth standards, using the Stata package zscore06.^15^ We reverse-coded age to estimate the risk of apnea associated with younger age and weight-for-age *z* score to estimate the risk of apnea associated with lower weight for age.

Sensitivity analyses adjusted (1) for corrected age^4^ (chronological age corrected for gestational age at birth) and weight-for-age *z* score and (2) for age, preterm birth, and birth weight less than 5 lbs (to convert to kilograms, multiply by 0.45). Given the possibility of variable collinearity, the variance inflation factor was calculated for all multivariable models. We also fit a locally weighted scatterplot smoothing (LOWESS) plot to show the association between serum albumin levels and the estimated probability of apnea from the multivariable model. All models accounted for potential patient clustering by site.

Final data analyses were conducted from February 25, 2019, to May 13, 2019, and used Stata, version 14.2 (StataCorp LLC). The comparison of RSV with non-RSV infection used a χ^2^ test. A 2-sided α = .05 was used to indicate statistical significance.

## Results

**Figure 1.**
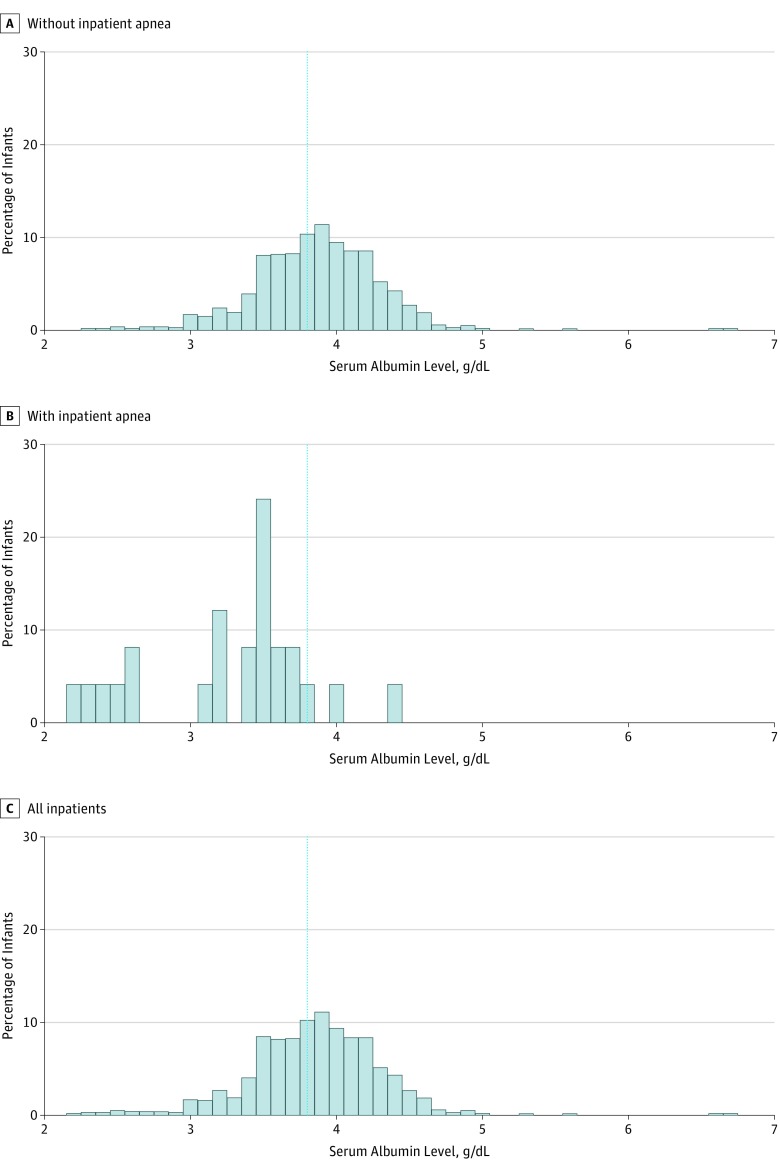
Distribution of Serum Albumin Levels Among Infants With or Without Apnea During Hospitalization The dotted blue line represents the serum albumin level categorized as normal (≥3.8 g/dL). To convert albumin level to grams per liter, multiply by 10.

Of the 1016 infants hospitalized for bronchiolitis, the median (interquartile range [IQR]) age was 3 (2-6) months, 610 (60.0%) were male, and 186 (18.3%) were born preterm (32-37 weeks’ gestation). The median (IQR) serum albumin level was 3.9 (3.6-4.1) g/dL, and 385 (37.9%) had low serum albumin levels. The prevalence of apnea among infants with a normal albumin level was 0.5% compared with a prevalence of 5.7% among all infants with a low albumin level and 14.6% among infants younger than 1 month with low albumin levels. Twenty-five infants (2.5%) had apnea while hospitalized, composing the cases for the primary analysis; among these 25 infants, the median (IQR) serum albumin level was 3.5 (3.1-3.6) g/dL, and 22 (88.0%) had low serum albumin levels. Figure 1 shows the distribution of albumin levels among infants with (n = 25) or without (n = 991) apnea during hospitalization. In addition to the 25 infants with apnea during their hospitalization (12 both before and during and 13 only during), 44 infants (4.3%) were apneic before but not during the hospitalization. These 44 infants were included in the secondary analysis of all apnea cases in the cohort (n = 69).

**Figure 2.**
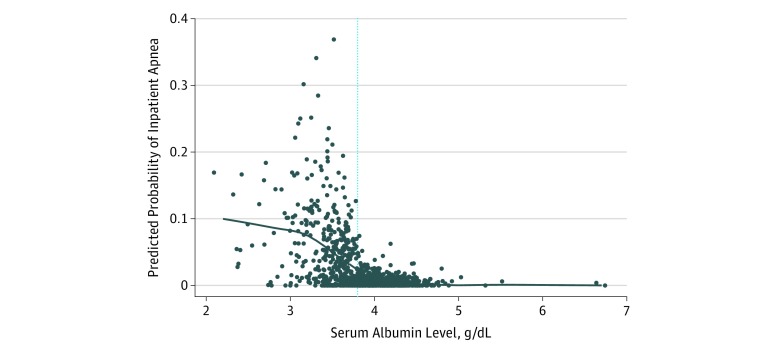
Locally Weighted Scatterplot Smoothing of Serum Albumin Level and the Estimated Probability of Apnea The dotted blue line represents the serum albumin level categorized as normal (≥3.8 g/dL). The predicted probability of inpatient apnea was obtained from a logistic regression model of serum albumin (<3.8 g/dL vs ≥3.8 g/dL) and weight-for-age *z* score. To convert albumin level to grams per liter, multiply by 10.

In unadjusted analyses (Table 1), low albumin level was associated with apnea (odds ratio [OR], 12.69; 95% CI, 3.23-49.82). As expected, younger age, preterm birth, and lower weight-for-age *z* score were all associated with apnea. However, when comparing infants with or without RSV infection, we found no difference in the prevalence of apnea (2.4% vs 2.6% *P* = .92). After adjustment for age, preterm birth, and weight-for-age *z* score, low serum albumin levels remained associated with a statistically significantly higher risk of apnea (OR, 4.42; 95% CI, 1.21-16.18; Table 1). The inverse association between serum albumin levels and estimated probability of apnea is shown in the LOWESS plot (Figure 2). Models adjusting for corrected age or birth weight (<5 lbs) yielded similar results. Calculation of variance inflation factor for all models did not suggest multicollinearity (all variance inflation factors <1.6). In the secondary analysis of all 69 infants with apnea, we observed an attenuated, but still significant, association between low serum albumin levels and apnea before and during hospitalization (OR, 2.50; 95% CI, 1.53-4.08; Table 2).

**Table 1.** Associations of Clinical Variables With Inpatient Apnea Among Infants Admitted for Bronchiolitis

Variable	Bivariate Models		Adjusted Model^a^	
	OR (95% CI)	*P* Value	OR (95% CI)	*P* Value
Serum albumin				
Low, <3.8 g/dL	12.69 (3.23-49.82)	<.001	4.42 (1.21-16.18)	.03
Normal, ≥3.8 g/dL	1 [Reference]	NA	1 [Reference]	NA
Younger age, mo	2.95 (1.68-5.21)	<.001	2.52 (1.34-4.72)	.004
Preterm birth, 32-37 wk	2.15 (0.73-6.32)	.17	1.82 (0.59-5.67)	.30
Lower weight-for-age *z* score	1.71 (1.33-2.20)	<.001	1.32 (0.99-1.77)	.06

Abbreviations: NA, not applicable; OR, odds ratio.

SI conversion factor: To convert albumin level to grams per liter, multiply by 10.

^a^ Multivariable logistic regression models were adjusted for age at hospitalization; preterm birth, defined as 32 to 37 weeks’ gestation; and weight-for-age *z* score at hospitalization.

**Table 2.** Associations of Clinical Variables With Apnea Before or During Hospitalization Among Infants Admitted for Bronchiolitis

Variable	Bivariate Models		Adjusted Model^a^	
	OR (95% CI)	*P* Value	OR (95% CI)	*P* Value
Serum albumin				
Low, <3.8 g/dL	4.14 (2.29-7.49)	<.001	2.50 (1.53-4.08)	<.001
Normal, ≥3.8 g/dL	1 [Reference]	NA	1 [Reference]	NA
Younger age, mo	1.43 (1.20-1.70)	<.001	1.28 (1.11-1.48)	.001
Preterm birth, 32-37 wk	2.23 (1.23-4.05)	.008	1.39 (0.72-2.67)	.32
Lower weight-for-age *z* score	1.93 (1.57-2.37)	<.001	1.65 (1.28-2.13)	<.001

Abbreviations: NA, not applicable; OR, odds ratio.

SI conversion factor: To convert albumin level to grams per liter, multiply by 10.

^a^ Multivariable logistic regression models were adjusted for age at hospitalization; preterm birth, defined as 32 to 37 weeks’ gestation; and weight-for-age *z* score at hospitalization.

## Discussion

In a large, prospective, multicenter cohort of infants hospitalized for bronchiolitis, low serum albumin levels were associated with increased risk of apnea after adjustment for known apnea risk factors (young age, preterm birth, and weight for age at hospitalization). Although needing replication and lacking a clear mechanism, these results suggest, for the first time to our knowledge, that albumin levels are a promising line of inquiry to help identify apnea in children hospitalized for bronchiolitis.

These results confirmed 2 important findings from a previous bronchiolitis cohort.^4^ First, in the previous severe bronchiolitis cohort, the viral origin of bronchiolitis was not associated with the risk of apnea.^4^ In the present cohort, compared with non-RSV infection, RSV bronchiolitis was not associated with higher risk of apnea. Second, in the previous severe bronchiolitis cohort, most children with a history of apnea did not have apnea while hospitalized. Specifically, 60% of children with a history of apnea did not have apnea in the hospital.^4^ In the present cohort, 44 (78.6%) of the 56 infants with a history of apnea did not have apnea in the hospital. This finding may be reassuring to families who have infants with a history of apnea, but a corollary of this result is that 13 (18.8%) of 69 infants with apnea in the present cohort only had apnea while in the hospital. Given that apnea does not always occur early in the clinical course of bronchiolitis^4^ and that many children who ultimately become apneic have no history of apnea, identifying an objective marker of apnea risk has the potential to change clinical practice and improve the quality of care for infants with bronchiolitis.

Although using albumin as a variable of apnea or as a component of an apnea prediction tool is promising, the distribution of albumin levels among infants with or without inpatient apnea shows many false-positives (94% of infants with low albumin levels did not have inpatient apnea) and a few false-negatives (0.5% of infants with normal albumin level had inpatient apnea). Until further research is completed, we do not recommend clinicians check albumin levels in infants with bronchiolitis unless otherwise clinically indicated. Replication of this finding in a larger cohort of infants is warranted.

Nonetheless, serum albumin levels may eventually play a role in identifying apnea in infants with severe bronchiolitis. Low serum levels of albumin, a multifunctional protein,^16^ have been associated with mortality in children and adults with multiple medical conditions, including respiratory illnesses.^7,8,9,10^ The present results extend this association with mortality to apnea, a life-threatening complication of bronchiolitis. The mechanism of low albumin levels in bronchiolitis is uncertain. One possibility is decreased synthesis of albumin. Although poor nutrition is commonly considered the main origin of hypoalbuminemia, not all malnourished individuals have low albumin levels.^17^ The present results show that the association between albumin and apnea is independent of weight-for-age *z* score. Furthermore, because the half-life of albumin is approximately 3 weeks,^18^ infants younger than 1 month with low albumin levels would be malnourished from birth. The multivariable models, however, suggest that the association between albumin and apnea is independent of low birth weight. Thus, although decreased synthesis of albumin from poor nutrition may be part of the mechanism of low serum albumin levels in bronchiolitis, it is not the only factor. Another possible mechanism of low serum albumin would be increased losses in the urine or stool, or increased breakdown of albumin. In the present cohort, we did not measure urine or stool albumin levels and have not conducted serum amino acid analysis to suggest increased albumin breakdown.

Another potential mechanism for the low serum albumin levels observed in this cohort is the inflammatory process associated with viral infections, specifically neurogenic inflammation. Piedimonte and colleagues^19^ have demonstrated in animal models that RSV via nerve growth factor increases nociceptive fibers; substance P; and 1 of its receptor subtypes, neurokinin 1.^20^ Of particular relevance to the association between albumin and apnea is that, with the use of labeled albumin in RSV-inoculated F-344 rats, substance P mediated the extravasation of albumin into the airways.^19,21,22^ What remains unclear is why neurogenic inflammation and albumin extravasation exist in the rare cases of apnea compared with other infants with severe bronchiolitis. Different virus-host interactions, virus microbiome, or even differing viral gene sequences all potentially exist and require further research.^11,23,24,25^

### Limitations

This study has several limitations. First, the apnea cases were identified by medical record review. Although it was possible that these infants were not truly apneic, the prevalence of inpatient apnea (2.5%) was within the range of previous prevalence estimates. Moreover, diagnosis in the hospital setting (rather than at home) made it more likely that these infants were truly apneic. Second, the mechanism for low albumin levels in bronchiolitis with apnea remains uncertain, despite the intriguing animal data about neurogenic inflammation. Third, given the small number of apnea outcomes in the hospitals, sparse data bias was likely.^26^ However, when we extended the apnea cases to those that occurred both before and during the hospitalization, the association between low serum albumin levels and apnea remained significant. Fourth, the association between albumin and apnea may not be generalizable beyond the study population of infants hospitalized for bronchiolitis. These results, however, would apply to the approximately 130 000 infants hospitalized with bronchiolitis annually.^1^

## Conclusions

In a large, prospective, multicenter cohort of infants hospitalized for bronchiolitis, low serum albumin level appeared to be associated with increased risk of apnea after adjustment for known apnea risk factors (young age, preterm birth, and weight-for-age *z* score). Although in need of replication, this finding may help inform and encourage future efforts to anticipate apnea, a life-threatening complication of bronchiolitis.
